# Dissociation and Isomerization Following Ionization of Ethylene: Insights from Nonadiabatic Dynamics Simulations

**DOI:** 10.1021/acs.jpca.3c06512

**Published:** 2024-02-15

**Authors:** Lina Fransén, Thierry Tran, Saikat Nandi, Morgane Vacher

**Affiliations:** † Nantes Université, CNRS, CEISAM UMR 6230, F-44000 Nantes, France; ‡ Université de Lyon, Université Claude Bernard Lyon 1, CNRS, Institut Lumière Matière, F-69622 Villeurbanne, France

## Abstract

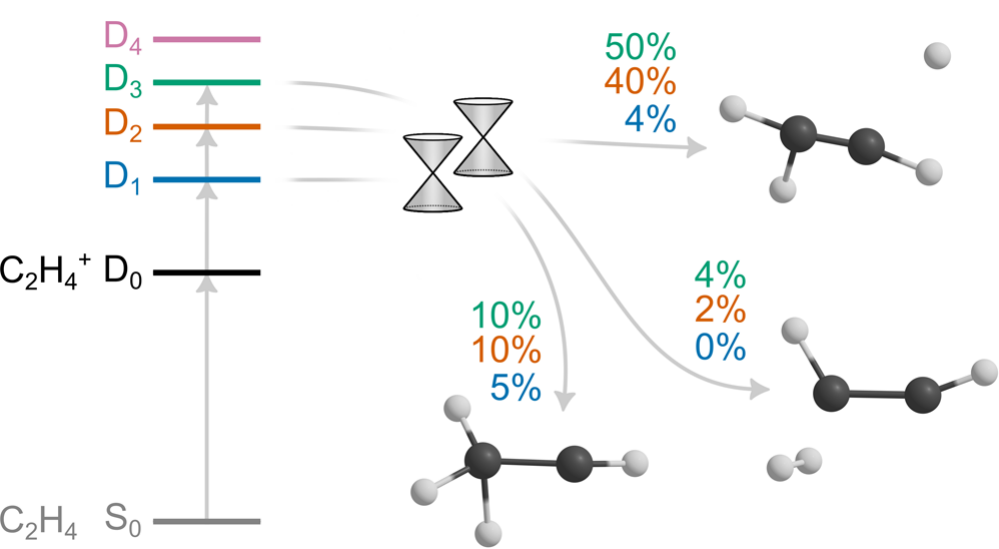

Photoionized and electronically excited ethylene C_2_H_4_^+^ can undergo H-loss, H_2_-loss, and ethylene–ethylidene isomerization, where the latter entails a hydrogen migration. Recent pioneering experiments with few-femtosecond extreme ultraviolet pulses and complementary theoretical studies have shed light on the photodynamics of this prototypical organic cation. However, no theoretical investigation based on dynamics simulations reported to date has described the mechanisms and time scales of dissociation and isomerization. Herein, we simulate the coupled electron–nuclear dynamics of ethylene following vertical ionization and electronic excitation to its four lowest-lying cationic states. The electronic structure is treated at the CASSCF level, with an active space large enough to describe bond breaking and formation. The simulations indicate that dissociation and isomerization take place mainly on the cationic ground state and allow the probing of previous hypotheses concerning the correlation between the photochemical outcome and the traversed conical intersections. The results, moreover, support the long-standing view that H_2_-loss may occur from the ethylidene form. However, the ethylene–ethylidene isomerization time predicted by the simulations is considerably longer than those previously inferred from indirect experimental measurements.

## Introduction

The ethylene cation, the simplest organic π radical, has served as a prototype system in experimental and theoretical investigations on the mechanism of electronic relaxation, structural dynamics, vibronic coupling, and energy redistribution.^1−13^ Dynamics simulations have predicted that electronically excited C_2_H_4_^+^ relaxes to the lowest-lying doublet state through conical intersections (CIs) associated with twisted and planar geometries,^8,10,11^ and dissociation via H- and H_2_-loss has been demonstrated experimentally.^1^ Elimination of H_2_ has, based on static simulations, been suggested to proceed via isomerization to the ethylidene form CH_3_CH^+^.^5^

A number of recent experimental works have utilized the time resolution afforded by emerging technologies to investigate the nonadiabatic dynamics of photoionized and electronically excited ethylene.^9−13^ Zinchenko et al. observed by attosecond transient absorption spectroscopy that electronic relaxation between the low-lying states of C_2_H_4_^+^ takes place in less than 7 fs.^11^ Several experimental^9^ and combined experimental-theoretical^10,12,13^ investigations on this system have moreover employed XUV-pump NIR-probe schemes, where the pump is an attosecond pulse train generated by high-harmonic generation (HHG),^14^ and the probe is a replica of the HHG driving pulse. The spectrally broad XUV pump ionizes neutral ethylene and launches a nuclear wave packet on several cationic electronic states simultaneously; the probe provides indirect information on the ensuing relaxation dynamics by inducing molecular fragmentation. Challenges associated with this experimental protocol include (i) disentangling the many parallel relaxation pathways and (ii) rationalizing the time-dependent fragment ion yields. Complementary theoretical studies, and in particular, nonadiabatic dynamics simulations, have proved to be useful in the interpretation of the experimental data.^10,12,13^ Recently, a comparison between an experimental and a simulated isotope effect was shown to aid the identification of the electronic states and nuclear coordinates involved in the relaxation process.^12^

Considerable research effort has been devoted to understanding the mechanisms of ethylene–ethylidene isomerization, H-loss, and H_2_-loss. Using the XUV-pump NIR-probe scheme described above, van Tilborg et al. inferred experimentally an ethylene–ethylidene isomerization time of 50 ± 25 fs.^9^ This isomerization time was later refined, also experimentally, to 30 ± 3 fs by Ludwig et al., who employed an experimental setup with higher time resolution.^10^ No statistically significant amount of H- or H_2_-loss was predicted by the TD-DFT/PBE0 nonadiabatic dynamics simulations supplementing the experiments in Ludwig et al.,^10^ and ethylene–ethylidene isomerization was not discussed either from a theoretical point of view therein. Similarly, Joalland et al. note in their purely theoretical work, where the electronic structure was treated at the complete active space self-consistent field (CASSCF) level with active spaces comprising 11 electrons in 7 orbitals and 11 electrons in 8 orbitals, that “No dissociation events have been observed from the excited states, nor any H migrations, although dissociation might be possible with a more flexible wave function (i.e., with a larger active space).”^8^

Joalland et al.,^8^ nevertheless, hypothesized factors that may govern the competition between H- and H_2_-loss. They identified two distinct classes of CIs, characterized by planar and twisted geometries. The population transfer through the latter class was shown to be enhanced at high excitation energies. To rationalize an experimentally observed decrease in the H_2_-/H-loss ratio at high excitation energies,^7^ Joalland et al. therefore proposed that the twisted relaxation channel may suppress ethylene–ethylidene isomerization (and by extension H_2_-loss) by inhibiting the required vibrational energy redistribution; transitions at twisted geometries were associated with an excitation of the vibrational mode ν_4_, which, because of symmetry, may not mix with the other modes. A planar CI located along the H-migration coordinate was, conversely, proposed to favor ethylene–ethylidene isomerization and H_2_-loss. These hypotheses are in contradiction with the views of Lorquet et al. and Sannen et al., who based on static calculations proposed that excitation of ν_4_ is required for ethylene–ethylidene isomerization,^4^ and that the competition between H- and H_2_-loss is governed by a CI along a C–H stretching coordinate.^5^

The details of the dissociation and isomerization dynamics of the ethylene cation and the influence of the CIs on this photoreactivity are still not well understood. In this work, we simulate the dissociation and isomerization dynamics of ethylene following ionization and excitation to its four lowest-energy cationic electronic states using surface hopping, a mixed quantum-classical nonadiabatic dynamics method. The electronic structure is described at the CASSCF level, with an active space comprising all valence electrons (11 electrons in 12 orbitals). The large active space allows a description of bond breaking, and thereby an exploration of the mechanisms, yields, and time scales of H-loss, H_2_-loss, and ethylene–ethylidene isomerization. The simulation results moreover enable a revisiting of previous hypotheses concerning the photochemical outcomes favored by relaxations through twisted and planar CIs.

This article is organized as follows. The theoretical methods are detailed in the next section. The subsequent section presents the results and discusses the findings in the context of previously published experimental and theoretical works. The last section then concludes the article.

## Theoretical Methods

All electronic structure and nonadiabatic dynamics calculations were carried out using the OpenMolcas^15,16^ software (version 22.10-354-g7f2c128^17^).

### Electronic Structure

The electronic structure of the ethylene cation was treated with the CASSCF method^18^ with state-averaging over the five lowest-energy cationic states. An active space comprising 11 electrons in 12 orbitals was employed: as shown by some of the present authors, this active space is large enough to describe bond breaking and formation.^12^ The following orbitals were included: the σ and σ* orbitals of the four C–H bonds, and the σ, σ*, π, and π* orbitals of the C=C bond (see Figure S1 in the Supporting Information). Only the C 1s orbitals were thus inactive. The effect of adding dynamic electron correlation through XMS-CASPT2 was tested with scans along key coordinates (Figures S2–S4 in the Supporting Information): in general, no significant differences were observed. The atomic compact Cholesky decomposition^19^ was used to reduce the computational cost, and the basis set of choice was the atomic-natural-orbital relativistic with core correlation basis set with polarized double-ζ contraction (ANO-RCC-VDZP).^20^

Minimum energy conical intersections (MECIs) were optimized at the above-cited level of theory starting from ∼150 D_1_/D_0_ hopping geometries from the dynamics simulations as well as from previously reported structures.^8^ Moreover, the neutral ground state was optimized and associated harmonic frequencies were calculated using an active space of 12 electrons distributed in 12 orbitals. Here, only the lowest-energy root was considered.

### Nonadiabatic Dynamics

Initial conditions were generated using Newton-X^21^ by sampling 300 geometries and velocities in an uncorrelated fashion from the Wigner distribution^22^ using the frequencies computed at the optimized neutral ground state. For each of the 300 pairs of geometries and velocities, one trajectory was initiated on each of the D_1_, D_2_, and D_3_ states. 100 trajectories were initiated on D_0_. This approach treats the electronic superposition as an incoherent one.

Nonadiabatic dynamics simulations were carried out using the surface hopping method employing the Tully fewest switches algorithm.^23^ Hops between the five lowest-energy cationic states were allowed. The trajectories were propagated with a nuclear time step of 20 au (0.48 fs) for ∼200 fs (203 fs); adequate energy conservation with this time step is demonstrated in Figure S5 in the Supporting Information. Each nuclear time step was split into 96 substeps for the propagation of the electronic wave function, which was performed with the Hammes-Schiffer Tully scheme^24^ using biorthonormalization, as recently implemented in OpenMolcas.^25^ The energy-based decoherence correction devised by Granucci and Persico^26^ was employed, with a decay factor of 0.1.

### Analysis

A small number of trajectories, amounting to 0.0, 0.3, 3.3, and 7.3% of those initiated on D_0_, D_1_, D_2_, and D_3_, respectively, exhibit a change in total energy of more than 0.5 eV during the dynamics. These trajectories were discarded, and the analyses include the remaining 967 trajectories. A statistical ensemble is essential for exploring the relationship between the photochemical outcome and the CI that mediated the electronic decay.

The norms of the Dyson orbitals, which provide estimates of the ionization intensities, are relatively independent of the initial geometry (see Figure S6 in the Supporting Information). Ensemble properties were therefore computed as unweighted averages of all trajectories. Uncertainties associated with the ensemble size were estimated with the bootstrap method using 1000 resamples.

The trajectories with exactly three (instead of four) H atoms closer than 2.9 Å (the sum of the van der Waals radii of H and C) to their nearest C atom are classified as having undergone H-loss. Similarly, those with exactly two H atoms closer than 2.9 Å to their nearest C atom are taken as H_2_- or 2H-loss trajectories. Trajectories with all four H atoms at a distance of less than 2.9 Å from their nearest C atom, and with three H atoms closer to one C atom than to the other, are considered to be in the ethylidene form. We note that this geometrical criterion differs from that employed in ref (8). The structures termed therein the “bridged minimum” and the “ethylidene geometry” (where the latter was identified as a transition state) would in the present work both be classified as ethylidene: for both structures, three H atoms are closer to one of the C atoms than to the other. The fraction of trajectories in the ethylidene form is defined as the ethylidene isomer population.

To investigate the correlation between the photochemical outcome and the CI that mediated the electronic decay to the cationic ground state, the D_1_→D_0_ hopping geometries were clustered with the optimized D_1_/D_0_ MECI structures. This was done by computing the RMSDs of the former geometries relative to the latter using the RMSD Python package.^27^ The pairs of Cartesian coordinates were aligned using the Kabsch algorithm^28^ for removing rotation and translation. Mirroring was also considered in the minimization of the RMSD. The trajectories were assigned according to the smallest RMSD.

## Results and Discussion

This section starts with a description of the ionization/excitation scheme and the electronic population decays. Following this, an overview of the simulated dissociation and isomerization dynamics is provided. The remainder of the section is then concerned with exploring the correlation between the photochemical outcome and the CIs mediating the preceding electronic decay. In pursuance of this, the excitation-energy dependence of the branching between planar and twisted relaxation pathways is reexamined, and optimized D_1_/D_0_ MECIs are discussed.

### Ionization and Excitation, and Ensuing Electronic Relaxation

**Figure 1:**
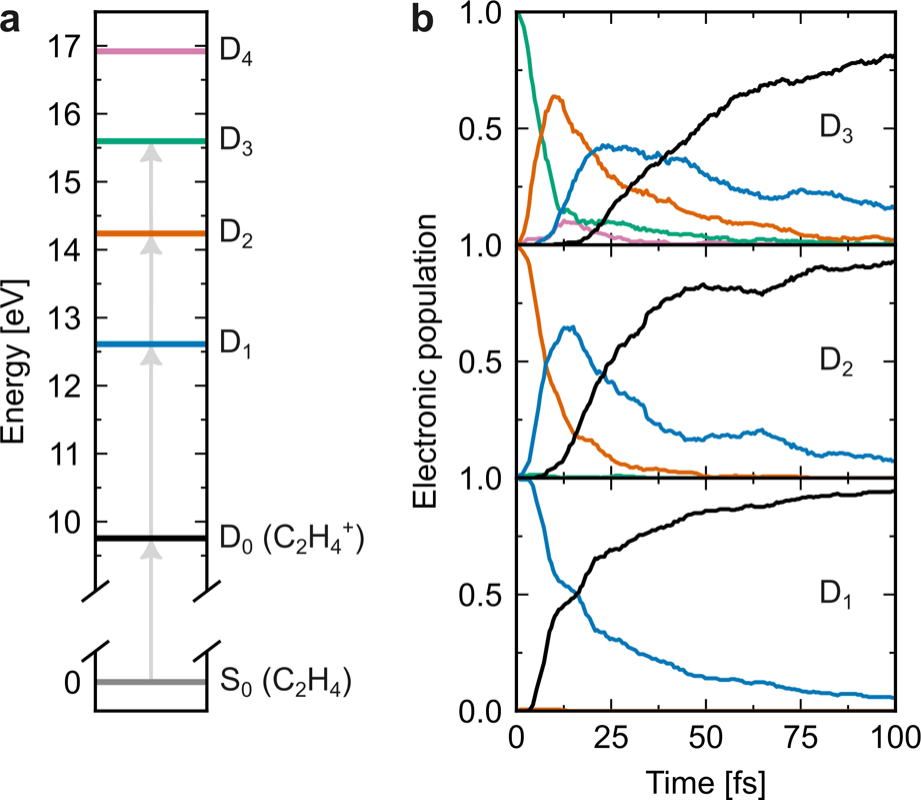
(a) Ionization and excitation of neutral ethylene in its ground electronic state (S_0_) to the four lowest-energy cationic electronic states (D_0_–D_3_). The horizontal lines show the computed energies of D_0_–D_4_ at the Franck–Condon point (the optimized geometry of the neutral ground state). (b) Time-dependent adiabatic electronic populations following ionization and electronic excitation to D_3_ (top), D_2_ (middle) and D_1_ (bottom).

This work simulates the dynamics upon photoionization and electronic excitation of neutral ethylene to the four lowest-energy cationic states D_0_–D_3_ (Figure 1a). The photoelectron spectrum computed for these transitions (Figure S7 in the Supporting Information) shows good agreement with a previously reported experimental one.^29^ The cationic ground state is characterized by a singly occupied π orbital, and D_1_–D_3_ are all characterized by singly occupied σ orbitals. Several recent experimental investigations employed pump–probe schemes in which the pump ionizes and excites neutral ethylene to one^11^ or several^9,10,12,13^ of these electronic states, allowing a comparison between theory and experiment.

Figure 1b shows the time evolution of the adiabatic electronic state populations. The electronic relaxation is ultrafast: 50% of the population has decayed to D_0_ within 48, 25, and 16 fs following excitation to D_3_, D_2_, and D_1_, respectively. The D_1_ → D_0_ electronic relaxation time determined herein is somewhat slower than that reported in the work of Zinchenko et al.,^11^ where an experimental D_1_ → D_0_ decay time of 6.8 ± 0.2 fs was obtained by attosecond transient absorption spectroscopy at the carbon K-edge. The experimental observable, which is not directly comparable with the adiabatic electronic decay, was simulated by AIMS combined with X-ray spectroscopic calculations. Nevertheless, we consider the presently calculated time scale to be in qualitative agreement with this measurement and to be within the precision range expected for the surface hopping method. Indeed, the electronic population decay can depend on the scheme used to approximate the nonadiabatic couplings.^25^

### Dissociation and Isomerization: Yields and Time Scales

**Figure 2:**
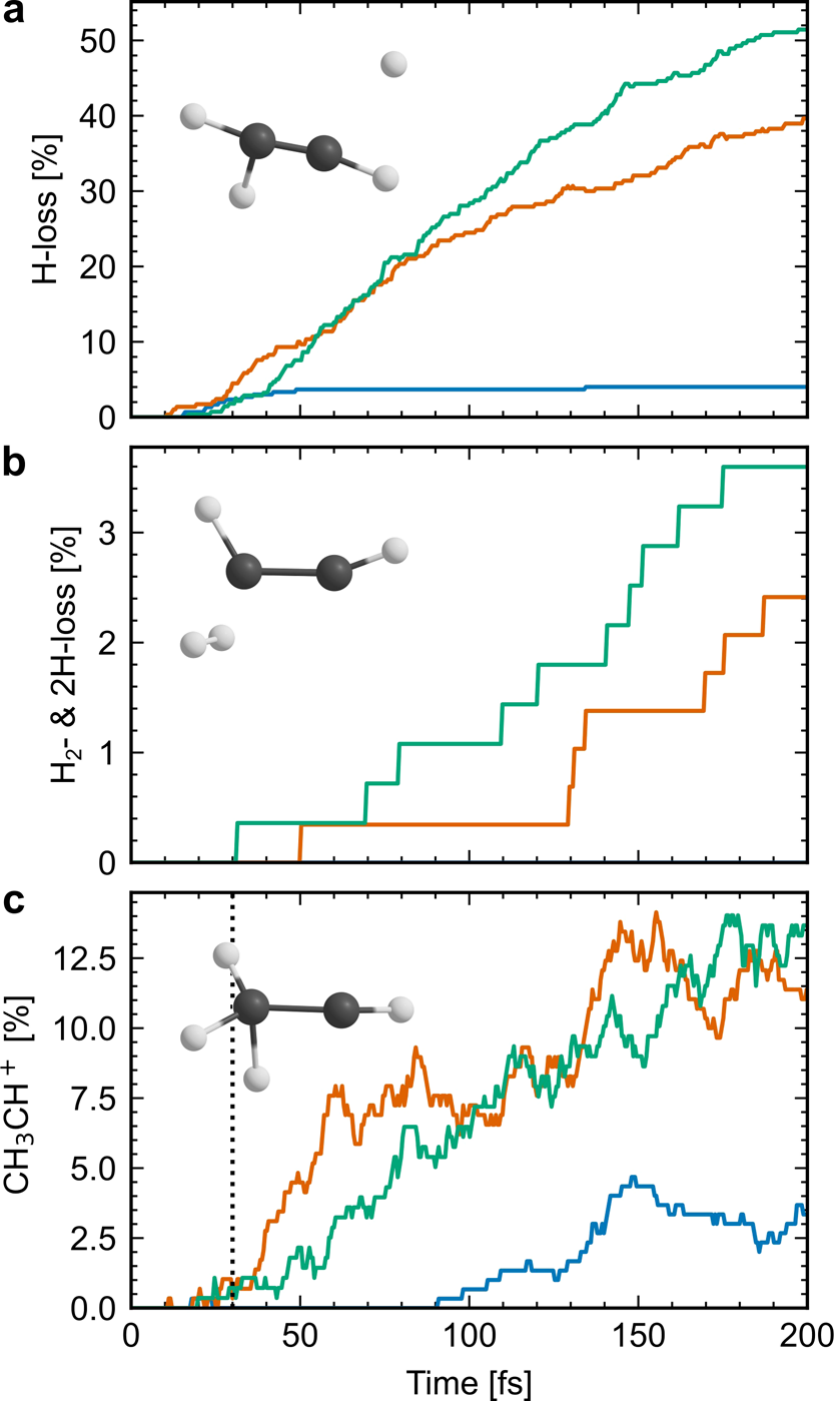
(a) Time-dependent H-loss yields following initial population of D_1_ (blue), D_2_ (orange) and D_3_ (green). (b) Same as panel a, but for H_2_- and 2H-loss. (c) Same as panel a, but for the ethylidene isomer population. The vertical dashed line indicates a previously experimentally inferred ethylene–ethylidene isomerization time from ref (10).

In terms of nuclear dynamics, a significant amount of C–H bond dissociation and ethylene–ethylidene isomerization is observed in the simulations. These photochemical events take place almost exclusively after the ultrafast electronic relaxation to D_0_ (see Table S1 in the Supporting Information). The time evolution of the H-loss yield, the H_2_- and 2H-loss yield, and the ethylidene isomer population are shown in Figure 2a–c, respectively. The results are displayed separately for ionization and excitation to D_1_, D_2_, and D_3_. The contributions from excitation to each of D_0_–D_3_ to the simulated photoelectron spectrum (Figure S7 in the Supporting Information) are largely nonoverlapping, making a grouping of the trajectories according to the initial state roughly equivalent with a grouping according to the excitation energy. None of the trajectories initiated on D_0_ dissociates or isomerizes, and no H_2_- or 2H-loss is observed for those initiated on D_1_. In the following, the time scale and mechanism of each photochemical channel are discussed in more detail.

The time-dependent H-loss yields reported in Figure 2a are defined as the percentage of the trajectories that have exactly three H atoms attached to a C atom at a given time step (see Theoretical Methods for details). At the end of the simulation time (200 fs), ∼40 and ∼50% of the trajectories initiated on D_2_ and D_3_, respectively, have undergone H-loss. The results are qualitatively different upon D_1_ excitation, where the yield saturates at only 4% after ∼50 fs.

The simulations thus indicate a strong excitation-energy dependence of the H-loss yield, in line with experimental data reported by Lucchini et al.^13^ They employed an XUV-pump NIR-probe scheme and varied the composition of the initial electronic superposition by selecting single harmonics. Approximate H-loss yields can be extracted from a graph in ref (13) displaying static (XUV-pump only) C_2_H_3_^+^ ion yields. These were ∼10% upon excitation with H9, which populates mainly D_0_ and D_1_, and ∼40% upon excitation with H11 and H13, which populate D_2_ and D_3_ more efficiently. Note that the above-cited experimental values are the asymptotic (long-time) yields, and that the presently modeled H-loss yields may increase beyond the simulation time window.

In addition to the experimental results described above, Lucchini et al. reported mixed nonadiabatic and adiabatic dynamics simulations with 4, 41, and 55% H-loss upon excitation to D_1_, D_2_, and D_3_, respectively.^13^ The first part of the dynamics was described with the surface hopping method with a CASSCF(11e,9o) treatment of the electronic structure. According to the authors, this active space is not suited to describe fragmentation because of the limited number of active orbitals. They, therefore, switched to adiabatic dynamics at the DFT/B3LYP level of theory after relaxation to D_0_. The adiabatic dynamics were propagated for 2 ps, but the time scale and mechanism of H-loss was not described. In the present work, a statistically significant amount of H-loss is predicted despite maintaining the same electronic structure method throughout the nonadiabatic dynamics simulations, which we attribute to the larger CASSCF active space.

Corresponding data for the competing H_2_- and 2H-loss channel is shown in Figure 2b. On the time scale of the simulations, 0, 2.4, and 3.6% of the population initiated on D_1_, D_2_, and D_3_, respectively, undergo H_2_- or 2H-loss. These yields are approximately 1 order of magnitude lower than the experimental C_2_H_2_^+^ ion yields extracted from data reported by Lucchini et al. (∼20% for all three harmonics^13^). To explain this discrepancy, we hypothesize that H_2_- and 2H-loss continues on a time scale exceeding the simulated 200 fs. The amount of H_2_-loss from the ethylidene isomer may depend on the cumulative time spent in the latter form, which keeps increasing (Figure 2c). In the mixed nonadiabatic and dynamics simulations of Lucchini et al. (discussed above), 1, 16, and 13% H_2_- and 2H-loss were reported 2 ps after excitation to D_1_, D_2_, and D_3_, respectively.^13^ However, as was the case for H-loss, the time scale and mechanism of H_2_- or 2H-loss were not discussed therein.

Three distinct H_2_- and 2H-loss mechanisms are represented in the simulations of the present work (Table 1): (I) H_2_-loss from the ethylidene isomer, (II) sequential 2H-loss from ethylene, and (III) H_2_-loss from ethylene. Note here that the branching ratios between them reported in Table 1 should be interpreted with care because of the limited statistics.

**Table 1:** Mechanisms of H_2_- and 2H-Loss and the Branching Ratios between Them Following Ionization and Excitation to D_2_ and D_3_^a^

	mechanism	D_2_	D_3_
I	CH_2_CH_2_^+^ → CH_3_CH^+^ → CHCH^+^ + H_2_	86% (6/290)	40% (4/278)
II	CH_2_CH_2_^+^ → CH_2_CH^+^ + H → CHCH^+^ + 2H	0% (0/290)	20% (2/278)
III	CH_2_CH_2_^+^ → CCH_2_^+^ + H_2_	14% (1/290)	40% (4/278)

a The numbers of trajectories corresponding to the percentages are indicated in parentheses.

H_2_-loss from the ethylidene isomer dominates following ionization and excitation to D_2_. Upon initial population of D_3_, H_2_-loss from ethylidene and ethylene contribute equally, with a lesser contribution from 2H-loss from ethylene. To our knowledge, only mechanism (I) has been discussed previously in the literature in the context of the ultrafast dynamics of the ethylene cation, and this mechanism has previously only been supported by static calculations. 2H-loss exhibits a higher threshold from the cationic ground state (7.3 eV^1^) compared to H_2_-loss (2.6 eV^1^), and it is therefore reasonable that, if the former contributes, it does so only upon high-energy excitation. These three mechanisms cannot be distinguished by experimental schemes relying solely on the C_2_H_2_^+^ fragment as a signature for H_2_- or 2H-loss.

We now turn our attention to the ethylidene isomer, which is involved in H_2_-loss mechanism (I). Figure 2c shows the ethylidene isomer populations, defined as the percentage of the trajectories that are in the CH_3_CH^+^ form (see Theoretical Methods for definition) at a given time step. Ethylene–ethylidene isomerization is reversible on the time scale of the simulations, as indicated by the nonmonotonic increases in the populations in Figure 2c and as inferred by visual inspection of the time evolution of the nuclear geometries along selected trajectories using the molden program.^30^

The time-dependent CH_3_CH^+^ populations are rather similar for D_2_ and D_3_ excitation. They start to build up at ∼30 fs, and thereafter continue to increase until the end of the simulation time, where they reach ∼10–15%. The population increase following D_2_ excitation is more uneven than that following D_3_ excitation. Upon ionization and excitation to D_1_, the modeled onset of ethylene–ethylidene isomerization occurs later, at ∼90 fs, despite the faster electronic relaxation to D_0_. At ∼150 fs, a local maximum, at ∼5%, is thereafter seen.

The time scale of ethylene–ethylidene isomerization has previously been inferred from indirect experimental measurements employing XUV-pump NIR-probe schemes.^9,10^ In these works, the CH_3_^+^ ion—assumed to be formed by a probe-induced breakup of the C=C bond in CH_3_CH^+^—was taken as a signature of the isomerization. From a fit to the CH_3_^+^ ion yield, Ludwig et al. extracted an upper bound of the ethylene–ethylidene isomerization time of 30 ± 3 fs,^10^ which is indicated in Figure 2c by the dashed vertical line. They define it as “the time that the nuclear wave packet needs to reach a region of the potential energy surface where the isomer population probability is maximum.”^10^ As evident from Figure 2c, the present work’s modeled isomerization time differs considerably from the above cited experimentally inferred one: 30 fs after ionization and excitation to D_1_, D_2_, and D_3_ (the states mainly populated experimentally by the XUV-pump in ref (10)), the simulated ethylidene isomer population probabilities are essentially zero. This large discrepancy may indicate an alternative interpretation of the time dependence of the experimental CH_3_^+^ ion yield.

### Dissociation and Isomerization: Role of Conical Intersections

After having described the overall yields and time scales of H-loss, H_2_- and 2H-loss, and ethylene–ethylidene isomerization in the preceding subsection, the present subsection aims to explore if, and in that case how, these yields depend on the CIs that mediated the electronic decay to D_0_ (recall here that most dissociation and isomerization events take place on D_0_). As a first step, hopping geometries and D_1_/D_0_ MECIs are presented.

**Figure 3:**
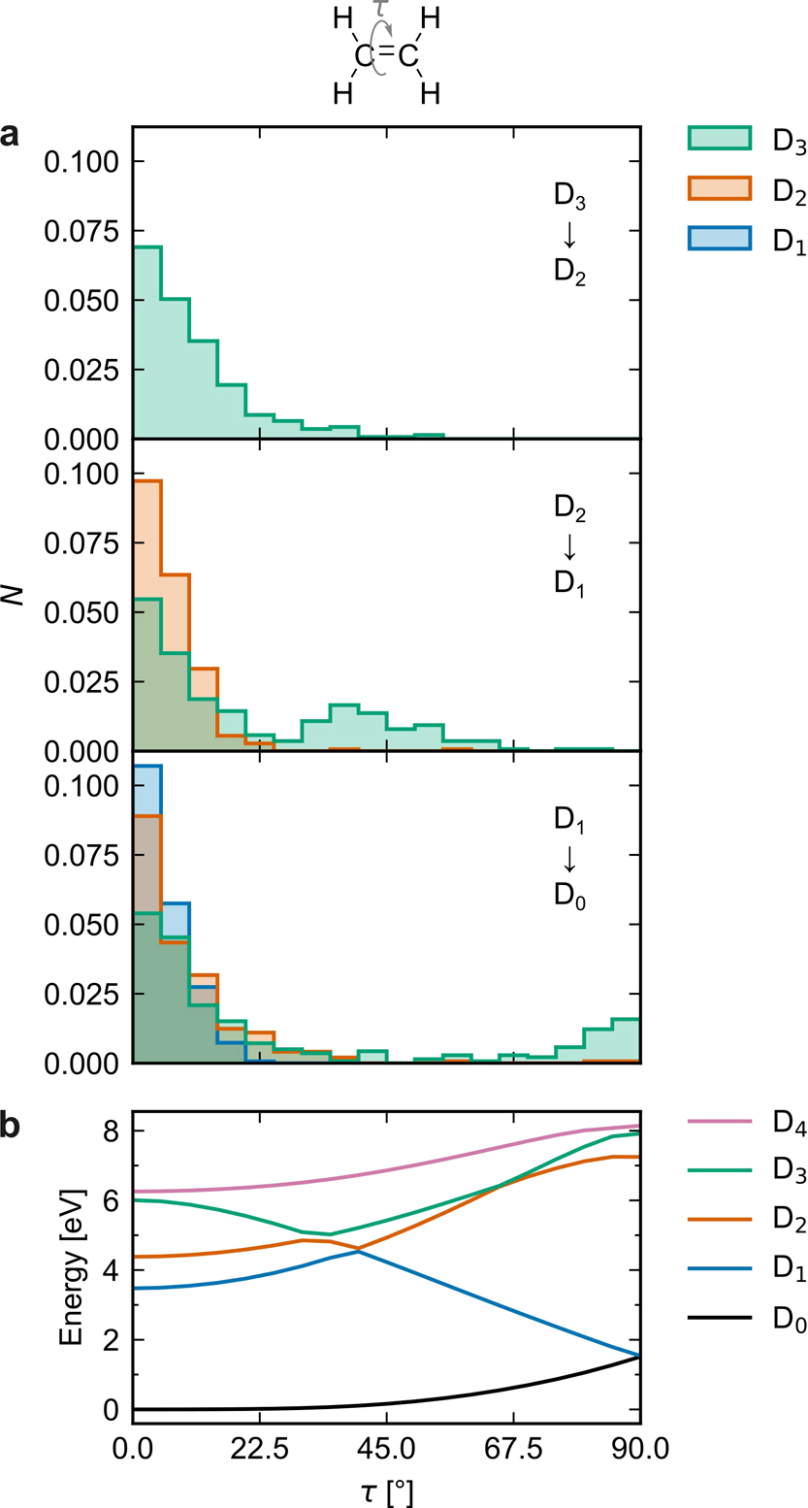
(a) Distributions of dihedral angles at the D_3_ → D_2_ (top), D_2_ → D_1_ (middle), and D_1_ → D_0_ (bottom) hopping geometries following excitation to D_1_ (blue), D_2_ (orange), and D_3_ (green). (b) Potential energy curves along the H–C–C–H dihedral coordinate τ obtained by constrained optimization on D_0_.

The nonadiabatic transitions between the low-lying states of C_2_H_4_^+^ have previously been shown to proceed via two distinct classes of CIs characterized by planar and twisted geometries.^8,10,13^ MECIs are presented in detail below, after a discussion of hopping geometries. Figure 3a shows the distribution of H–C–C–H dihedral angles τ for the D_3_ → D_2_, D_2_ → D_1_, and D_1_ → D_0_ hopping geometries upon initial population of D_1_, D_2_, and D_3_. The graphs display the dihedral angle at each trajectory’s first hop between the given pair of states (many trajectories undergo back-hopping and thereafter transition between the same pair of states again). Upon excitation to D_3_, the D_3_ → D_2_ transitions are centered around planar geometries (τ = 0°). The wavepacket then splits, with one part undergoing the D_2_ → D_1_ nonadiabatic transitions around planar, and the other around ∼45° twisted, geometries. Further twisting thereafter occurs, and the D_1_ → D_0_ transitions take place around planar and 90°-twisted geometries. In contrast, upon excitation to D_1_ and D_2_, the system is brought to D_0_ almost exclusively via planar CIs.

The torsional relaxation channel thus comes into play essentially only upon excitation to D_3_. This can be rationalized partly from the dihedral scan in Figure 3b, where the ∼45°-twisted D_2_/D_1_ and the 90°-twisted D_1_/D_0_ CIs are visible. The latter is a symmetry-induced (Jahn–Teller^31^) intersection. Around τ = 0°, where the Franck–Condon (FC) point is located upon vertical ionization and excitation of neutral ethylene, torsional barriers are present on D_1_ and D_2_, but not on D_3_. In agreement with our simulations, twisted CIs were involved almost exclusively upon D_3_ excitation in previous nonadiabatic dynamics simulations initiated around the equilibrium geometry of the neutral species.^10,13^ In ref (8), where the dynamics were initiated around the optimized geometry of the cation (|τ| = 18°), the twisted decay pathway contributed also upon D_1_ and D_2_ excitations, albeit to a lesser extent than after photoexcitation to D_3_.

In the following, we focus on the D_1_/D_0_ CIs and their effect on the ensuing dissociation and isomerization dynamics. Optimized D_1_/D_0_ MECIs and associated population transfers are first described, whereafter MECI-specific dissociation and isomerization yields are presented.

**Figure 4:**
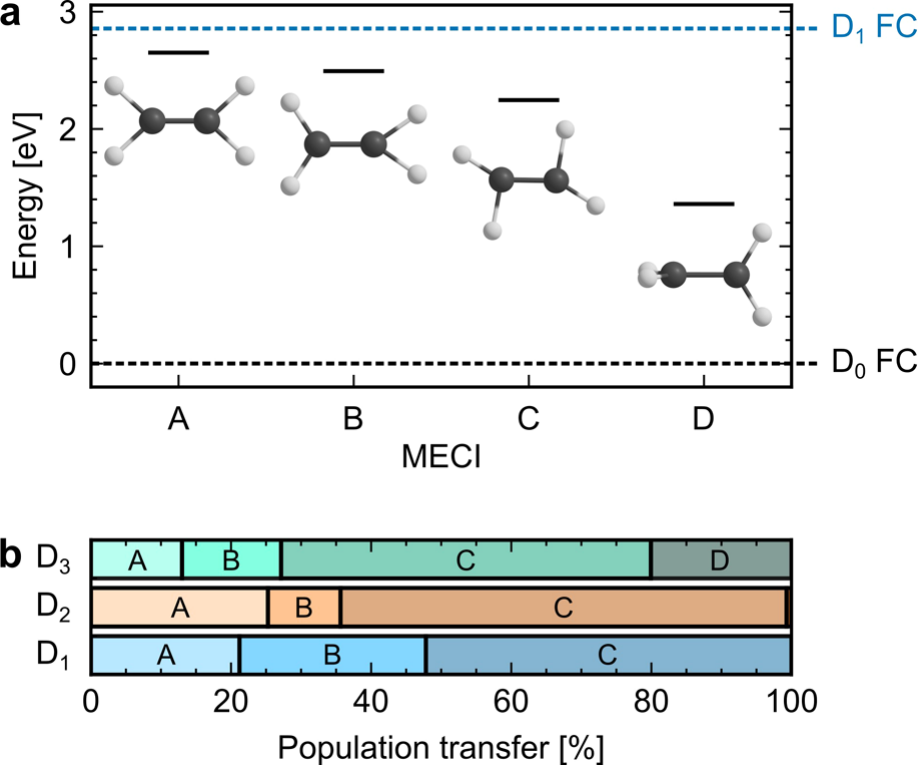
(a) Optimized D_1_/D_0_ MECIs. (b) Population transfers for each of the D_1_/D_0_ MECIs after excitation to D_3_ (top), D_2_ (middle) and D_1_ (bottom).

Four D_1_/D_0_ MECIs were identified, of which three are planar (A–C in Figure 4a) and one twisted (D in Figure 4a). All of them are energetically accessible from the D_1_ FC point. In terms of molecular geometry, MECI A is accessed by a contraction of the C=C bond and a symmetric scissoring of the H–C–H angles, and MECI B by asymmetric H–C–H scissoring. The two lowest-energy structures are C, located along an H-migration coordinate, and D, the 90°-twisted CI. MECIs B–D were previously described in ref (8), and a structure similar to A was identified in ref (11). A geometrical and energetic comparison of the presently found and previously reported MECIs is provided in Figure S8. In addition, CIs along a C–H stretch coordinate have been proposed to promote H-loss.^5,10^ Whereas we found points along this coordinate that are located on, or close to, the CI seam, our optimizations did not converge on minimum energy structures characterized by one or several elongated C–H bonds.

To obtain the population transfer associated with each MECI, the D_1_→D_0_ hopping geometries were clustered with structures A–D as described in the Theoretical Methods section. Upon excitation to D_1_, the hops to D_0_ take place close to the MECIs, as indicated by the narrow distribution of RMSDs (see Figure S9 in the Supporting Information). The D_1_ → D_0_ transitions are less tight following D_2_ and D_3_ excitations. This, coupled with the geometrical proximity of structures A–C, implies that the nonadiabatic transitions may occur almost equally close to several of these planar MECIs. Moreover, all optimizations initiated from ∼150 D_1_→D_0_ hopping geometries converged on the lowest energy structures C and D. Although the trajectories clustered with each of the structures within the planar family thus may not be well-separated from each other, this approach does well in separating the trajectories transitioning close to the family of planar MECIs (A–C) from those hopping close to the twisted MECI (D).

The resulting population transfers for each MECI are displayed in Figure 4b. Regardless of the initially populated state, relaxation through the planar MECI C dominates the decay, with smaller contributions from hops close to structures A and B. These results contrast with those of Zinchenko et al., who discussed the ultrafast D_1_ → D_0_ decay upon ionization and excitation of neutral ethylene to D_1_ in terms of MECI A only.^11^ The torsional relaxation channel (MECI D) contributes almost exclusively upon D_3_ excitation, in agreement with the data reported in Figure 3a. The population transfers associated with MECIs B–D were previously reported in ref (8), but a direct comparison cannot be made due to the different initiation of the dynamics.

Next, we turn our attention to the MECI-specific isomerization and dissociation yields and time scales. To rationalize an experimentally observed decrease in the H_2_-/H-loss ratio at high excitation energies,^7^ Joalland et al. proposed that transition through twisted CIs may hamper ethylene–ethylidene isomerization and thereby disfavor H_2_-loss.^8^ The planar MECI C has, conversely, been proposed to promote ethylene–ethylidene isomerization.^8,10^

**Figure 5:**
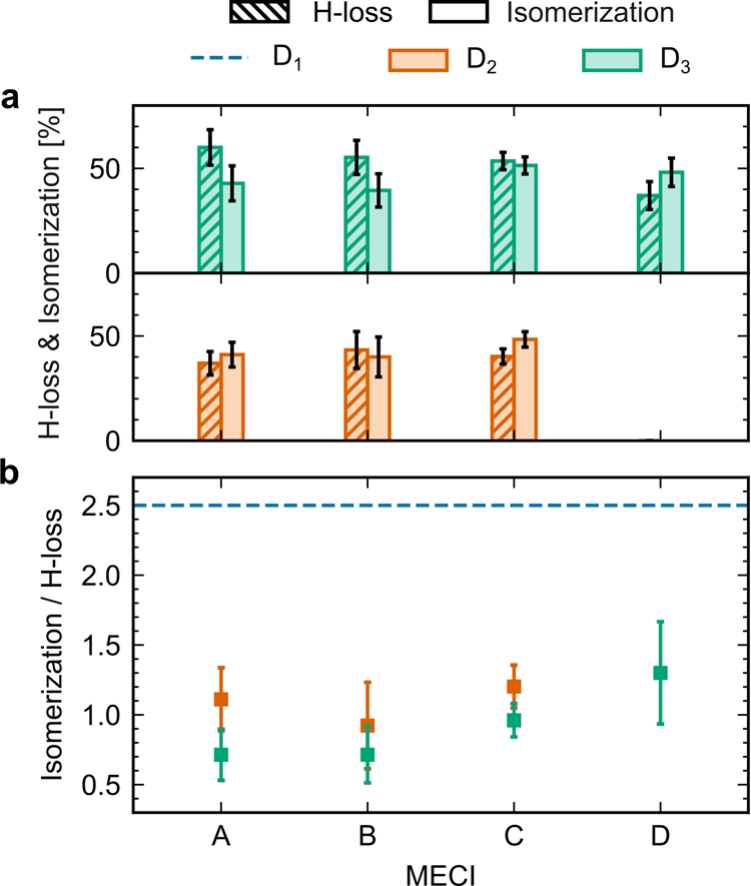
(a) D_1_/D_0_ MECI-specific H-loss and ethylene–ethylidene isomerization yields following ionization and electronic excitation to D_3_ (top) and D_2_ (bottom). The error bars show one standard deviation obtained by bootstrapping with 1000 resamples. (b) MECI-specific ethylene–ethylidene isomerization/H-loss ratios following D_3_ (green) and D_2_ (orange) excitation. The blue dashed line shows the overall (across all MECIs) ethylene–ethylidene isomerization/H-loss ratio upon excitation to D_1_. The error bars show one standard deviation obtained by bootstrapping with 1000 resamples; no uncertainty is indicated for the ratio upon D_1_ excitation because the bootstrapping yielded a highly skewed distribution.

Figure 5a shows the yields of H-loss and ethylene–ethylidene isomerization for the trajectories clustered with each of the D_1_/D_0_ MECIs. The ethylene–ethylidene isomerization yield is here defined as the percentage of the trajectories that are in the ethylidene form during at least one time step of the dynamics; as noted previously, ethylene–ethylidene isomerization is reversible. Given the small number of H_2_- and 2H-loss trajectories (only 17 out of 967 trajectories in total), the statistics for this dissociation channel are insufficient for a meaningful comparison of the MECI-specific yields. Similarly, the trajectories initiated on D_1_ are excluded from the analysis because of the low H-loss yield (12 H-loss trajectories out of 299 trajectories for this initial state). Following initial ionization and excitation to D_2_, only two trajectories transition through the twisted MECI D, and these are therefore also excluded from the analysis.

Following ionization and electronic excitation to D_2_, the ethylene–ethylidene isomerization and H-loss yields appear to vary only slightly among the planar MECIs A–C. Figure 5a indicates that the variation in the yields associated with the planar MECIs may be larger upon D_3_ excitation–but the statistics do not allow for clear conclusions. The similarity of the photochemical outcomes associated with the different planar MECIs A–C may partly be attributed to the geometrical proximity of the latter, combined with the relatively large RMSDs between the hopping geometries and the MECIs. Figure 5a suggests that the trajectories relaxing to D_0_ at twisted geometries are less inclined to undergo H-loss during the 200 fs simulation time than those transitioning at planar geometries. Ethylene–ethylidene isomerization, in contrast, does not appear to be hampered. As a result, the ethylene–ethylidene isomerization/H-loss ratio (Figure 5b) is higher for the trajectories clustered with MECI D than for those clustered with the planar MECIs A–C (although the difference is not statistically significant).

**Figure 6:**
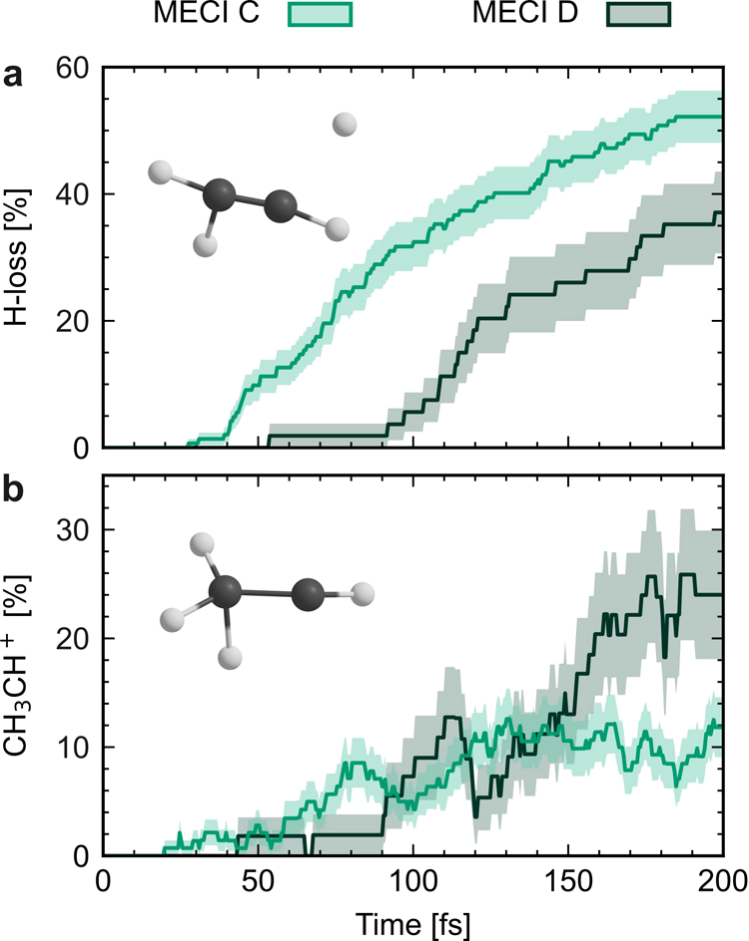
(a) Time-dependent H-loss yields for the trajectories transitioning through the planar D_1_/D_0_ MECI C (light green) and the 90°-twisted MECI D (dark green) following excitation to D_3_. The shaded regions show one standard deviation obtained by bootstrapping with 1000 resamples. (b) Same as (a), but for the ethylidene isomer population.

To gain further insight into the dissociation and isomerization dynamics associated with the planar and twisted relaxation pathways, the time evolution of the H-loss yields and CH_3_CH^+^ isomer populations are shown for MECI C and D in Figure 6. Later onsets of both ethylene–ethylidene isomerization and H-loss are observed for the trajectories transitioning at twisted geometries, which may in part be explained by a slower D_1_ → D_0_ decay. The slower decay is, in turn, mainly caused by an important amount of back-hopping; almost 90% of the trajectories that relax to D_0_ at twisted geometries hop back to D_1_ at least once. This feature has previously been reported in AIMS simulations.^8^ After the delayed onset, the ethylidene isomer population associated with MECI D increases, and at the end of the simulation time, it exceeds that associated with MECI C. The H-loss yield, on the other hand, remains lower throughout the simulation time.

The results presented in Figures 5 and 6 suggest that transitions through the 90°-twisted CIs do not hinder ethylene–ethylidene isomerization compared to H-loss, which contrasts with the hypothesis of Joalland et al. This indicates that the opening of the torsional relaxation channel may not be the origin of the experimentally observed^7,13^ decrease in the H_2_-/H-loss ratio at high excitation energies—although we note that the link between ethylene–ethylidene isomerization and H_2_-loss, on which this conclusion is based, is yet to be firmly established. The excitation-energy-dependent ethylene–ethylidene isomerization/H-loss ratio in Figure 5b may be in line with the experimentally observed excitation-energy-dependent H_2_-/H-loss ratios, if (i) the trend persists over time (and with increasing ensemble size), (ii) H_2_-loss following excitation to D_1_ occurs, like following D_2_ excitation, mainly from the CH_3_CH^+^ isomer, and (iii) the H_2_-loss yield correlates with the CH_3_CH^+^ isomer population.

## Conclusion

The present work used extensive surface hopping simulations to explore the mechanisms and time scales of H-loss, H_2_- and 2H-loss, and ethylene–ethylidene isomerization following photoionization and electronic excitation of ethylene. The electronic structure was treated at the CASSCF level with a large active space suited for a description of bond breaking and formation. The simulated H-loss yield shows a strong excitation-energy dependence, in agreement with previously reported experimental data.^13^ Further, in line with the mechanism proposed in the literature based on static calculations,^5^ the nonadiabatic dynamics simulations reported herein indicate that the C_2_H_2_^+^ fragment may be formed by H_2_-loss from CH_3_CH^+^. The simulations indicate further that two additional mechanisms—sequential 2H-loss from ethylene and H_2_-loss from ethylene—may contribute. We note, however, that the statistics for the H_2_- and 2H-loss channels are limited in the present work, and these results should therefore be interpreted with care. During the simulation time (200 fs), none of the trajectories initiated on D_1_ undergo H_2_- or 2H-loss, and the H_2_- and 2H-loss yields following D_2_ and D_3_ excitation are lower than previously reported experimental asymptotic (long-time) C_2_H_2_^+^ ion yields.^13^ Possibly, further H_2_- and 2H-loss occur on a time scale exceeding 200 fs.

The ethylene–ethylidene isomerization time predicted by the simulations is considerably longer than those inferred previously from time-dependent CH_3_^+^ ion yields from XUV-pump NIR-probe experiments.^9,10^ This raises questions about the interpretation of the experimental data. Previous studies relying on this experimental protocol indeed showed that the time-dependent fragment ion yields often are indirect signatures of the XUV-induced dynamics and that complementary dynamics simulations can aid the interpretation.^12,13^

The present paper moreover investigated the correlation between the photochemical outcome and the type of CI that mediated the preceding electronic decay (most dissociation and isomerization events take place on D_0_). In contrast to previous hypotheses,^8^ the simulations predict that when compared to transitions through the main planar D_1_/D_0_ CI located along the H-migration coordinate, transitions through the 90°-twisted CI do not suppress ethylene–ethylidene isomerization relative to H-loss. The results presented herein therefore suggest that the opening of the torsional relaxation channel at high excitation energies may not be responsible for the experimentally observed decrease in the H_2_-loss/H-loss ratio.^7,13^ This conclusion, however, relies on a link between ethylene–ethylidene isomerization and H_2_-loss, which is yet to be firmly established due to the limited statistics for the latter channel in the present work. Possibly in line with the above-cited experimental trend, the simulations indicate that the trajectories initiated on D_1_ may have a higher ethylene–ethylidene isomerization/H-loss ratio than those initiated on D_2_ and D_3_. This effect, however, cannot readily be traced back to different branching ratios between D_1_/D_0_ CIs.

Future simulations could address the long-time dissociation and isomerization dynamics of ionized and electronically excited ethylene, to see if the experimentally observed H_2_- and 2H-loss yield can be recovered at longer time scales. Such simulations may provide additional insight into the mechanism(s) of H_2_- and 2H-loss, and reveal whether the higher ethylene–ethylidene isomerization/H-loss ratio observed at low excitation energies in the present work translates into a higher H_2_/H-loss ratio.

## Supporting Information Available

Active space, effect of adding dynamic electron correlation, time step convergence, Dyson intensities and photoelectron spectrum, electronic state for dissociation and isomerization, comparison of MECIs, clustering of D_1_/D_0_ hopping geometries with MECIs, and optimized geometries (PDF)
