# Supplementary material for: Dissociation and Isomerization Following Ionization of Ethylene: Insights from Nonadiabatic Dynamics Simulations
Source: J Phys Chem A. 2024 Feb 15;128(8):1457–65. doi: 10.1021/acs.jpca.3c06512 (PMC10911106; doi:10.1021/acs.jpca.3c06512)
Supplement: Supplementary file 1 — jp3c06512_si_001.pdf [file jp3c06512_si_001.pdf]

Supporting Information: Dissociation and Isomerization Following Ionization of Ethylene: Insights from Non-adiabatic Dynamics Simulations

Lina Fransén,† Thierry Tran,† Saikat Nandi,‡ and Morgane Vacher*,†

†*Nantes Université, CNRS, CEISAM UMR 6230, F-44000 Nantes,France*

‡*Université de Lyon, Université Claude Bernard Lyon 1, CNRS, Institut Lumière Matière, F-69622, Villeurbanne, France*

E-mail: morgane.vacher@univ-nantes.fr

Active space

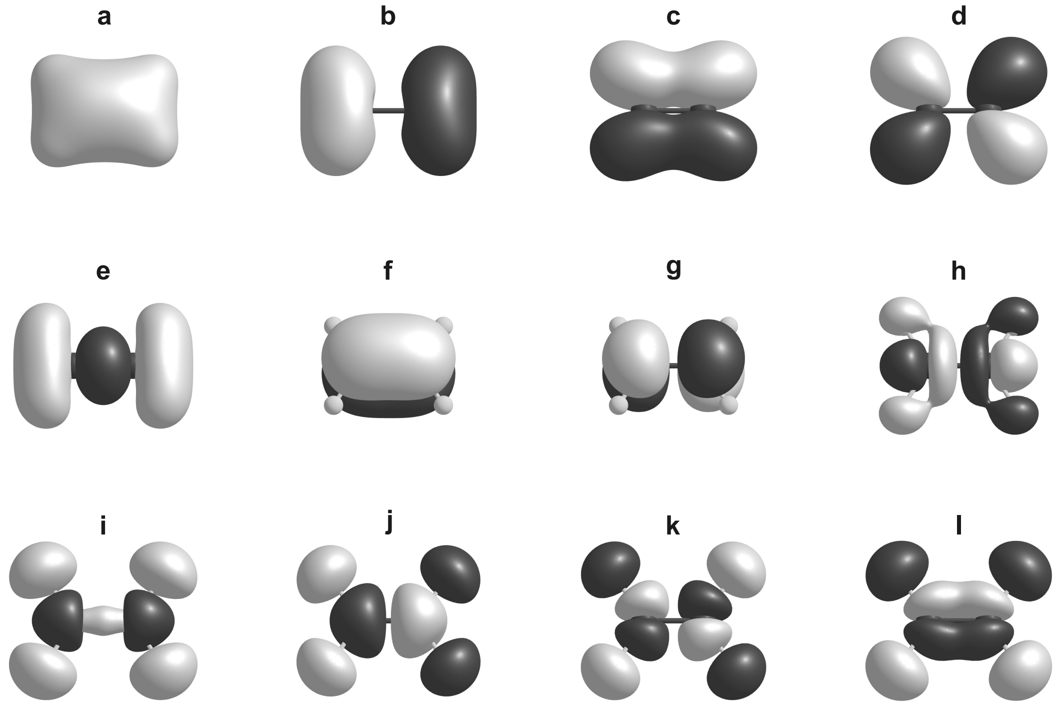

Figure S1: Orbitals included in the CASSCF active space: (a)-(d) C−H σ, (e) C−C σ, (f) C−C π, (g) C−C π^*, (h) C−C σ^*, (i)-(l) C−H σ^*.

Effect of adding dynamic electron correlation

A scan along the elongation of one of the C-H bonds (Figure S2a) suggests that the shape of the PES along this coordinate, which is here taken as characteristic of H-loss, is modified only slightly by the addition of dynamic electron correlation through XMS-CASPT2. Importantly, Figure S2a suggests that the barrier for H-loss on D_0 is well-described by CASSCF.

Figure S2b shows a scan along the H−C−C−H dihedral angle, which is a coordinate important to the electronic relaxation. Except for a reduction energy gap between the ground and the excited cationic states, the corrections provided by XMS-CASPT2 are minor.

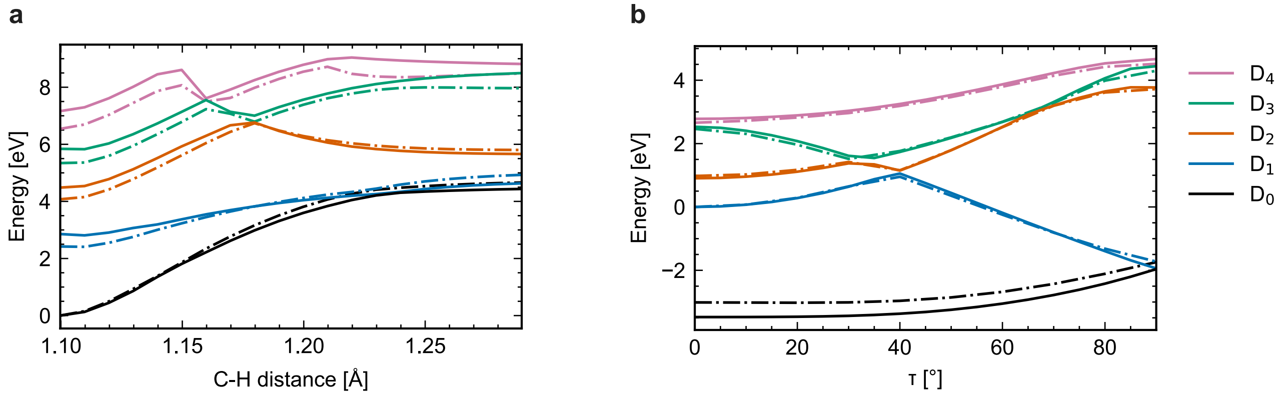

Figure S2: Comparison of SA5-CASSCF(11e/12o) (solid lines) and XMS-CASPT2//SA5-CASSCF(11e/12o) (dashed lines) along (a) the stretching of one C−H bond and (b) the H−C−C−H dihedral angle τ. An imaginary shift of $0.2\,E_\mathrm{h}$ was used for the XMS-CASPT2 calculations. The (a) D_0 and (b) D_1 energies provided by each method at the lower x-limit are set to 0.

XMS-CASPT2 energies for a few points along an example trajectory undergoing ethylene-ethylidene isomerization are shown in Figure S3. The corrections provided by XMS-CASPT2 are rather small (less than 0.27 eV for all structures).

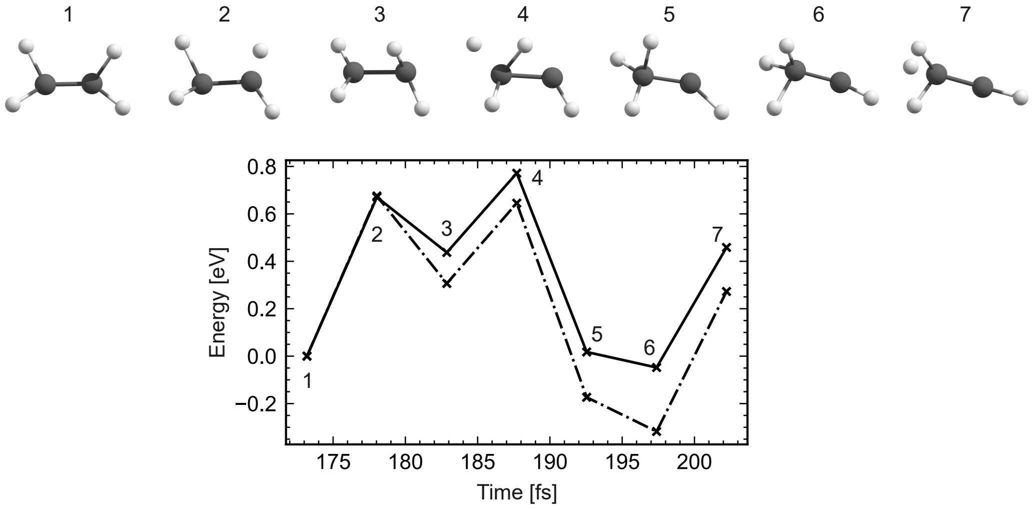

Figure S3: Comparison of SA5-CASSCF(11e/12o) (solid lines) and XMS-CASPT2//SA5-CASSCF(11e/12o) (dashed lines) along a trajectory undergoing ethylene-ethylidene isomerization. An imaginary shift of $0.2\,E_\mathrm{h}$ was used for the XMS-CASPT2 calculations. The energies provided by each method at point 1 are set to 0.

Figure S4 shows linear interpolation in internal coordinates (LIIC) scans between the Franck-Condon point and the three planar D_1/D_0 MECIs A-C. As was the case for the torsional scan in Figure S2b, the energy gaps between D_1 and D_0 are reduced upon the

addition of dynamic electron correlation through XMS-CASPT2. However, the shapes of the PESs are similar at the two levels of theory.

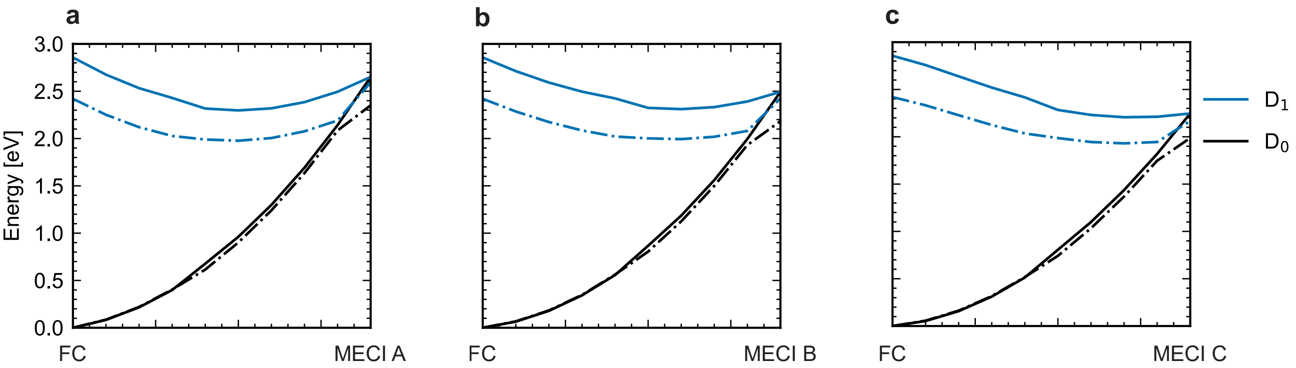

Figure S4: Linear interpolation in internal coordinates (LIIC) scans computed by SA5-CASSCF(11e/12o) (solid lines) and XMS-CASPT2//SA5-CASSCF(11e/12o) (dashed lines) between the Franck-Condon (FC) point and (a) MECI A, (b) MECI B, and (c) MECI C. An imaginary shift of $0.2\,E_\mathrm{h}$ was used for the XMS-CASPT2 calculations. The D_0 energies provided by each method at the FC point are set to 0.

Time step convergence

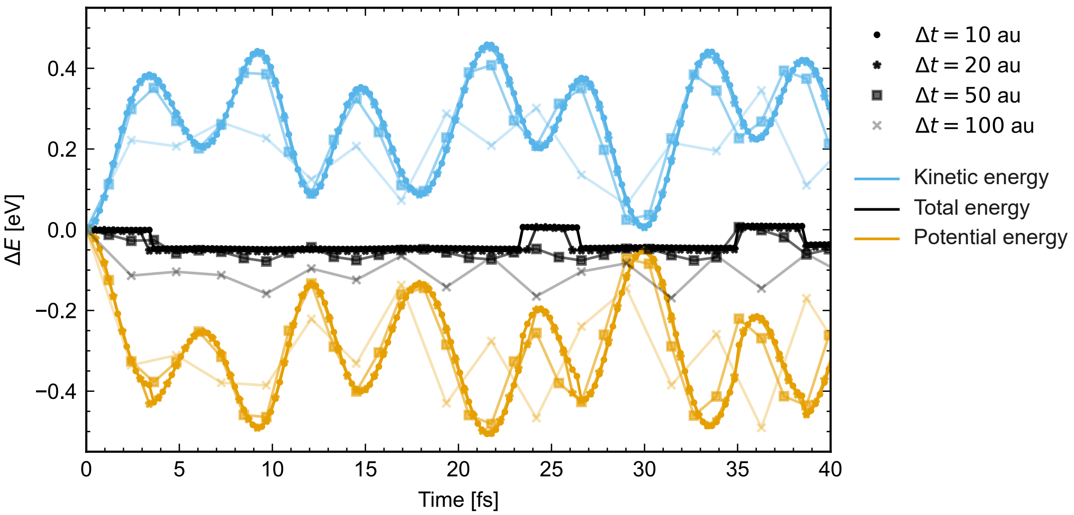

Figure S5: Conservation of energy for different nuclear time steps. The figure displays the time evolution of the potential, kinetic, and total energy of an "unsampled" trajectory initiated on D_1 at the optimized geometry of the neutral species with no kinetic energy.

Dyson intensities and photoelectron spectrum

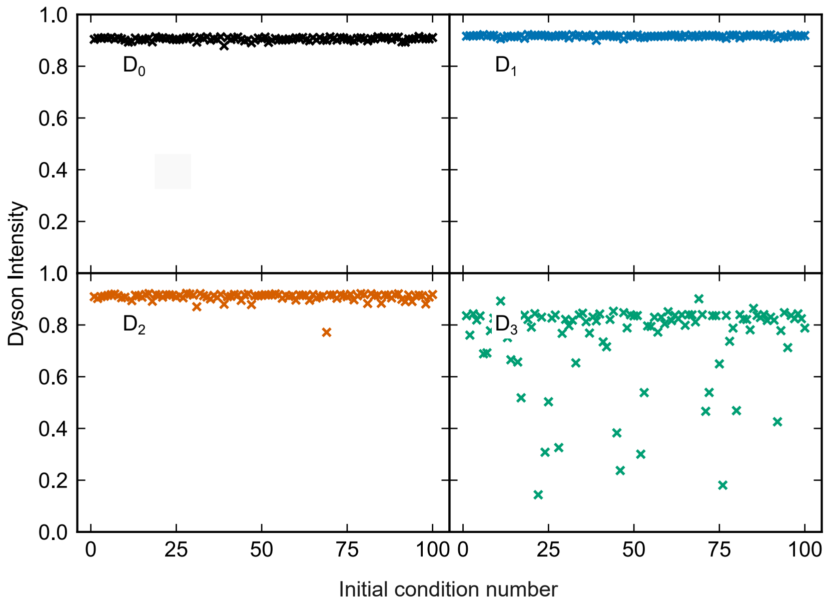

Figure S6: Dyson intensities for ionization to D_0, D_1, D_2 and D_3 for 100 of the initial conditions. For ionization to D_3, where the largest spread in Dyson intensities is observed, 75% of the initial conditions have Dyson intensities in the range 0.70-0.85.

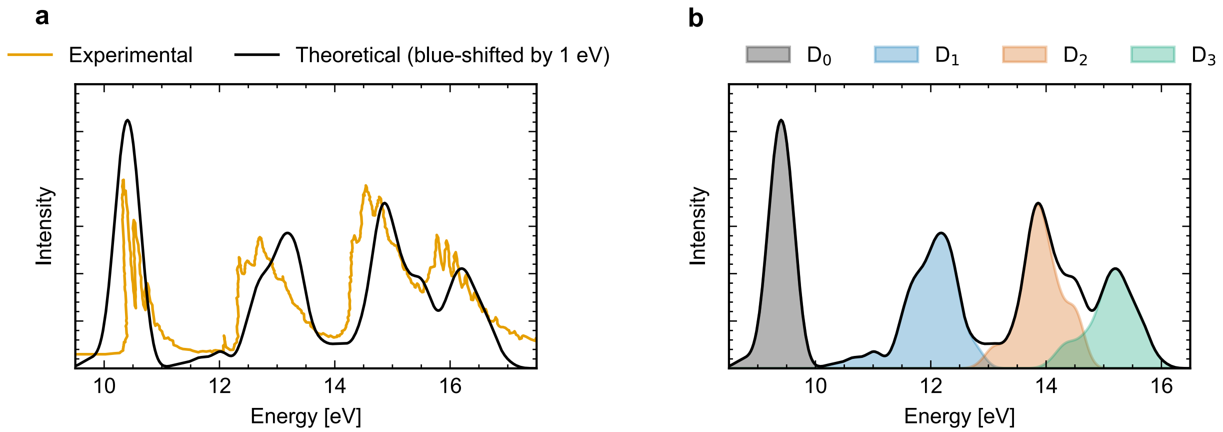

Figure S7: (a) Experimental photoelectron spectrum from Branton et al.,[S1] and spectrum simulated in the present work. The theoretical spectrum is blue-shifted by 1 eV to match the experimental one, and is based on the energies and squared Dyson norms for 100 initial conditions at $t = 0$. A Gaussian broadening with $\sigma = 0.2\,\text{eV}$ is applied. (b) Contributions from ionization and excitation to each of D_0-D_3 to the theoretical spectrum.

Electronic state for dissociation and isomerization

Table S1: Percentage of the dissociation and isomerization events that occur on D_0 following ionization and electronic excitation to D_1, D_2 and D_3. Each trajectory's first ethylene-ethylidene isomerization event is used for the statistics.

	H-loss [%]	H_2- and 2 H-loss [%]	Ethylene-ethylidene isomerization [%]
D_3	89	80	98
D_2	97	71	100
D_1	100	-	100

Comparison of MECIs

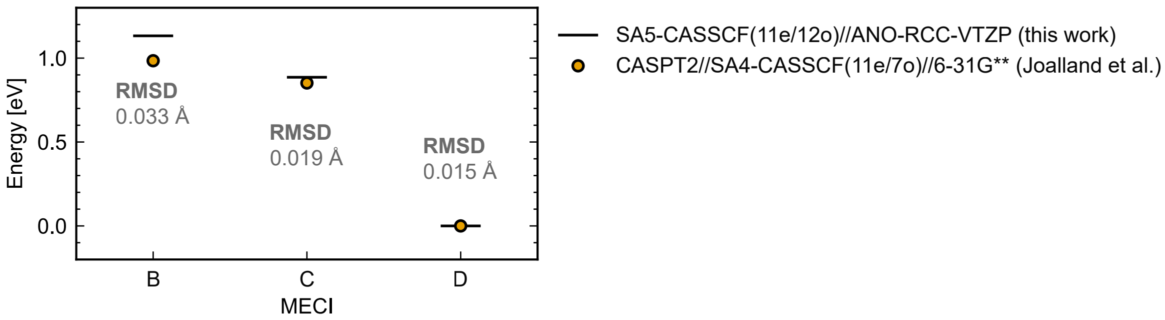

Figure S8: Comparison of the relative energies of the MECIs found in the present work and those reported by Joalland et al.[S2] The energies of the twisted MECI D in each work are taken as the reference points. The root-mean-square deviations (RMSDs) between the structures are also indicated.

In addition to the MECIs B-D previously reported by Joalland et al. (Figure S8), a structure similar to MECI A (see main text) has previously been described by Zinchenko et al. at the MR-CIS//3s2p1d(ANO) level of theory. The only structural information provided therein is the C-C bond length (1.251 Å) and the H−C−H angle (94.1°). MECI A in the present work has a slightly shorter C-C bond length (1.229) and a smaller H−C−H angle (85.0°).

Clustering of $D_1 \rightarrow D_0$ hopping geometries with MECIs

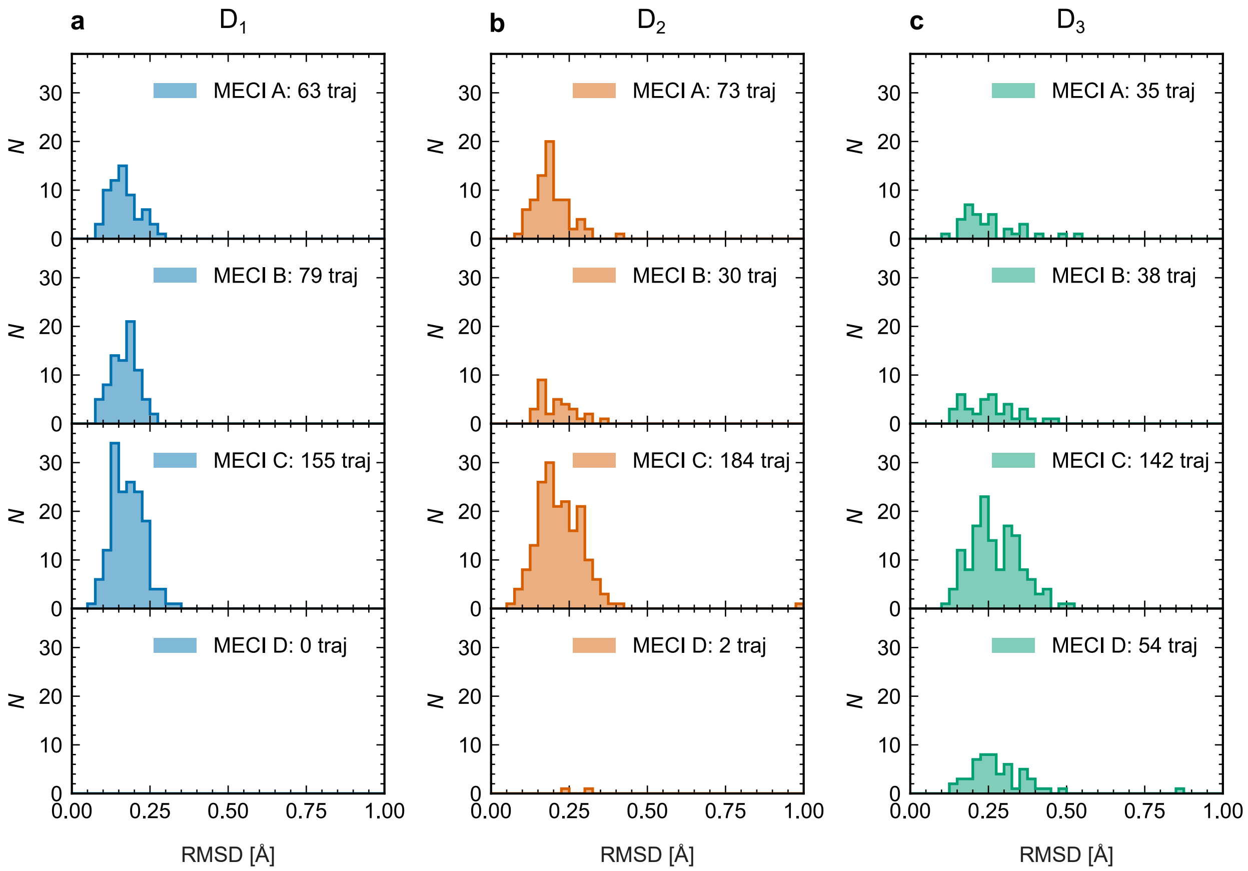

Figure S9: (a) $D_1 \rightarrow D_0$ hopping geometries following excitation to D_1 clustered with each of the MECIs A-D as a function of the RMSD from the relevant MECI. The graph includes each trajectory's first $D_1 \rightarrow D_0$ hopping geometry. (b) Same as a, but for excitation to D_2. (c) Same as a, but for excitation to D_3.

Optimized geometries

Coordinates (in Ångström) of the optimized S_0 ground state and the four D_1/D_0 MECIs.

```
  6
 S0 GS
C    -0.00000000     0.67505856    -0.00000000
C    -0.00000000    -0.67505856     0.00000000
H     0.00000000     1.25186548     0.93632930
H    -0.00000000     1.25186548    -0.93632930
H    -0.00000000    -1.25186548     0.93632930
H     0.00000000    -1.25186548    -0.93632930
```

```
  6
  D1/D0 MECI A
C     0.00000068     0.61459411    -0.00000020
C    -0.00000067    -0.61459172    -0.00000010
H    -0.00000012     1.47497378     0.78828736
H    -0.00000013     1.47497414    -0.78828721
H     0.00000012    -1.47497501     0.78828445
H     0.00000013    -1.47497530    -0.78828430
```

```
  6
  D1/D0 MECI B
C    -0.18766787     0.07962270     0.09559319
C     0.12758166     1.30520868     0.03446905
H    -0.69733333    -0.83127028    -0.47593495
H    -0.16400375    -0.90538590     0.76383366
H     0.65099914     1.84499954     0.86473269
H    -0.08982385     1.94763725    -0.85716164
```

```
  6
  D1/D0 MECI C
C    -0.32628197    -0.03810927     0.26533405
C     0.17353790     1.09083379    -0.09885606
H    -0.84846554    -0.36530605    -0.78457362
H    -0.47570150    -0.78339623     1.05675562
H     0.69542506     1.41757867     0.95144662
H     0.32305904     1.83675708    -0.88967861
```

```
  6
  D1/D0 MECI D
C    -0.00000051     0.71059255     0.00002186
C     0.00000051    -0.71059254     0.00002178
H    -0.67134163     1.29014384     0.67133325
H     0.67134181     1.29007882    -0.67135506
H     0.67134412    -1.29014377     0.67133076
H    -0.67134428    -1.29007889    -0.67135259
```

References

(S1) Branton, G. R.; Frost, D. C.; Makita, T.; McDowell, C. A.; Stenhouse, I. A. Photoelectron Spectra of Ethylene and Ethylene-d4. *The Journal of Chemical Physics* **1970**, *52*, 802–806.

(S2) Joalland, B.; Mori, T.; Martínez, T. J.; Suits, A. G. Photochemical Dynamics of Ethylene Cation ${C_2H_4}^+$. *The Journal of Physical Chemistry Letters* **2014**, *5*, 1467–1471.

(S3) Zinchenko, K. S.; Ardana-Lamas, F.; Seidu, I.; Neville, S. P.; van der Veen, J.; Lanfaloni, V. U.; Schuurman, M. S.; Wörner, H. J. Sub-7-Femtosecond Conical-Intersection Dynamics Probed at the Carbon K-edge. *Science* **2021**, *371*, 489–494.
